# An adherent-invasive *Escherichia coli*-colonized mouse model to evaluate microbiota-targeting strategies in Crohn's disease

**DOI:** 10.1242/dmm.049707

**Published:** 2022-10-24

**Authors:** Adeline Sivignon, Mélissa Chervy, Caroline Chevarin, Elia Ragot, Elisabeth Billard, Jérémy Denizot, Nicolas Barnich

**Affiliations:** ^1^ Université Clermont Auvergne, Inserm U1071, INRAE USC 1382, Microbes, Intestin, Inflammation et Susceptibilité de l'Hôte (M2iSH), Clermont-Ferrand 63001, France; ^2^ Institut Universitaire de Technologie, Génie Biologique, Aubière 63172, France

**Keywords:** AIEC, Ileal colonization, Mouse model, Crohn's disease, CEACAM6

## Abstract

Adherent-invasive *Escherichia coli* (AIEC) were investigated for their involvement in the induction/chronicity of intestinal inflammation in Crohn's disease (CD). AIEC gut establishment is favoured by overexpression of the glycoprotein CEACAM6 in the ileal epithelium. We generated a transgenic mouse model, named ‘Vill-*h*CC6’, in which the human *CEACAM6* gene was under the control of the villin promoter, conditioning expression in the small intestine. We demonstrated that CEACAM6 is strongly expressed in the small intestine mucosa and is correlated with numerous glycosylations displayed at the brush border of enterocytes. *Ex vivo*, the AIEC–enterocyte interaction was enhanced by CEACAM6 expression and necessitated the presence of the bacterial adhesive factor FimH. Finally, AIEC bacteria preferentially persisted in a FimH-dependent manner in the ileal mucosa of Vill-*h*CC6 mice compared to wild-type mice. This preclinical model opens new perspectives in the mechanistic study of the AIEC pathobiont and represents a valuable tool to evaluate the efficacy of new strategies to eliminate AIEC implanted in the ileal mucosa, such as phages, inhibitory and/or anti-virulence molecules, or CRISPR-based strategies targeting virulence or fitness factors of AIEC bacteria.

## Teaser

**Summary:** Adherent-invasive *Escherichia coli* (AIEC)-colonized transgenic mouse ‘Vill-*h*CC6’ reproducing ileal colonization by AIEC observed in Crohn's disease is proposed as a new tool to evaluate AIEC-targeting strategies.

## INTRODUCTION

Crohn's disease (CD) is an inflammatory bowel disease characterized by an abnormal immune response of the intestinal mucosa to environmental and bacterial triggers. To date, no curative treatment exists for CD patients, and current therapies only control the symptoms of the disease and aim to increase the time between relapses. It is therefore essential to identify the causes of the disease to develop more specific and personalized therapies. To date, the aetiology of CD is only partially understood, but it is clear that it results from complex interactions between environmental, genetic and microbial factors. Among the microbial factors identified as taking part in the onset and maintenance of the disease, a specific pathotype of *Escherichia coli* has been implicated, adherent-invasive *Escherichia coli* (AIEC) pathobiont bacteria, which are highly prevalent in ileal lesions of CD patients (Darfeuille-Michaud et al., 2004; Nadalian et al., 2021). We recently reported that AIEC encroachment to the ileal mucosa of CD patients is predictive of endoscopic postoperative recurrence at 6 months postsurgery, suggesting that AIEC-targeting therapeutic strategies could be of interest in the management of CD (Buisson et al., 2022). The adhesion ability of AIEC to intestinal epithelial cells (IECs) mainly relies on FimH adhesin, a mannose-binding lectin located at the tip of type 1 pili (Dreux et al., 2013; Boudeau et al., 2001). In the ileal mucosa of CD patients, carcinoembryonic antigen-related cell adhesion molecule 6 (CEACAM6) glycoprotein is abnormally expressed and harbours mannose residues recognized by bacterial FimH, thus favouring AIEC adhesion to IECs (Barnich et al., 2007). These bacteria play an important role in the induction and/or maintenance of intestinal inflammation through invasion of IECs, alteration of epithelial barrier integrity, and their ability to survive and replicate within macrophages, inducing secretion of proinflammatory cytokines such as tumor necrosis factor-α (TNF-α) (Chervy et al., 2020; Denizot et al., 2012; Sasaki et al., 2007; Douadi et al., 2022).

Based on these studies, many AIEC-targeting strategies are currently under investigation in preclinical models and in clinical trials to limit AIEC blooming and invasion of ileal mucosa. For example, the efficacy of antibiotics (TEOREM, NCT02620007) or phagotherapy (Galtier et al., 2017) (EcoActive, NCT03808103) to kill AIEC, or FimH antagonists (Sivignon et al., 2015b) (Sibofimloc, NCT03943446) and yeast probiotics (Sivignon et al., 2015a, 2021) to decrease AIEC gut colonization, are currently under study. However, a well-established and validated preclinical model closely mimicking AIEC-induced CD pathogenesis is required to validate new therapeutic agents targeting AIEC bacteria. To date, most published studies have used antibiotic-treated mice, dextran sulfate sodium (DSS)-induced colitis or genetically susceptible mouse models to reach AIEC colonic colonization (Spalinger et al., 2022; Carvalho et al., 2008; Bretin et al., 2018). The mouse genome lacks a human *CEACAM6* gene orthologue, making it difficult to reproduce the AIEC–CEACAM6 interaction *in vivo*. In previous studies, we used the humanized carcinoembryonic antigen bacterial artificial chromosome 10 (CEABAC10) mouse model, in which we showed that AIEC bacteria are able to colonize the colonic mucosa of mice in a CEACAM6/FimH-dependent manner and to induce an inflammatory response (Chan and Stanners, 2004; Carvalho et al., 2009; Lucchini et al., 2021; Sivignon et al., 2021). CEABAC10 mice carry a transgene encoding four genes of the CEACAM family members, including the *CEACAM6* gene. Of note, the transgene used in this mouse model is under the control of the human promoter, directing the expression of *CEACAM6* to colonic epithelial cells only, in which *CEACAM6* is physiologically expressed in humans. However, CD patients abnormally express CEACAM6 in the ileal mucosa, making CEABAC10 a suboptimal model for studying AIEC encroachment to the ileum (Barnich et al., 2007). These observations led us to drive human *CEACAM6* gene expression in small intestinal IECs to develop a new humanized mouse model that closely mimics the physiopathology of ileal CD.

In this work, we describe a new mouse model named ‘Vill-*h*CC6’, in which *CEACAM6* expression is driven by the mouse villin [also known as villin 1 (*Vil1*)] gene promoter, reproducing abnormal overexpression of the mannosylated human receptor CEACAM6 in the small intestine and allowing ileal colonization by AIEC bacteria, as observed in CD patients. Here, we present *ex vivo* and *in vivo* AIEC–enterocyte interaction tests and show that CEACAM6 overexpression in small intestine enterocytes correlates with high glycosylation of intestinal mucosa, favouring AIEC adhesion. We also validated our model using a type 1 pili-deficient AIEC strain, which shows decreased AIEC interaction with IECs. This model will be useful for screening and evaluating innovative strategies targeting the gut microbiota and, more specifically, the AIEC pathobiont, one possible trigger of intestinal inflammation.

## RESULTS

### Human CEACAM6 is highly expressed in the mucosa of the small intestine in Vill-*h*CC6 mice

**Fig. 1.**
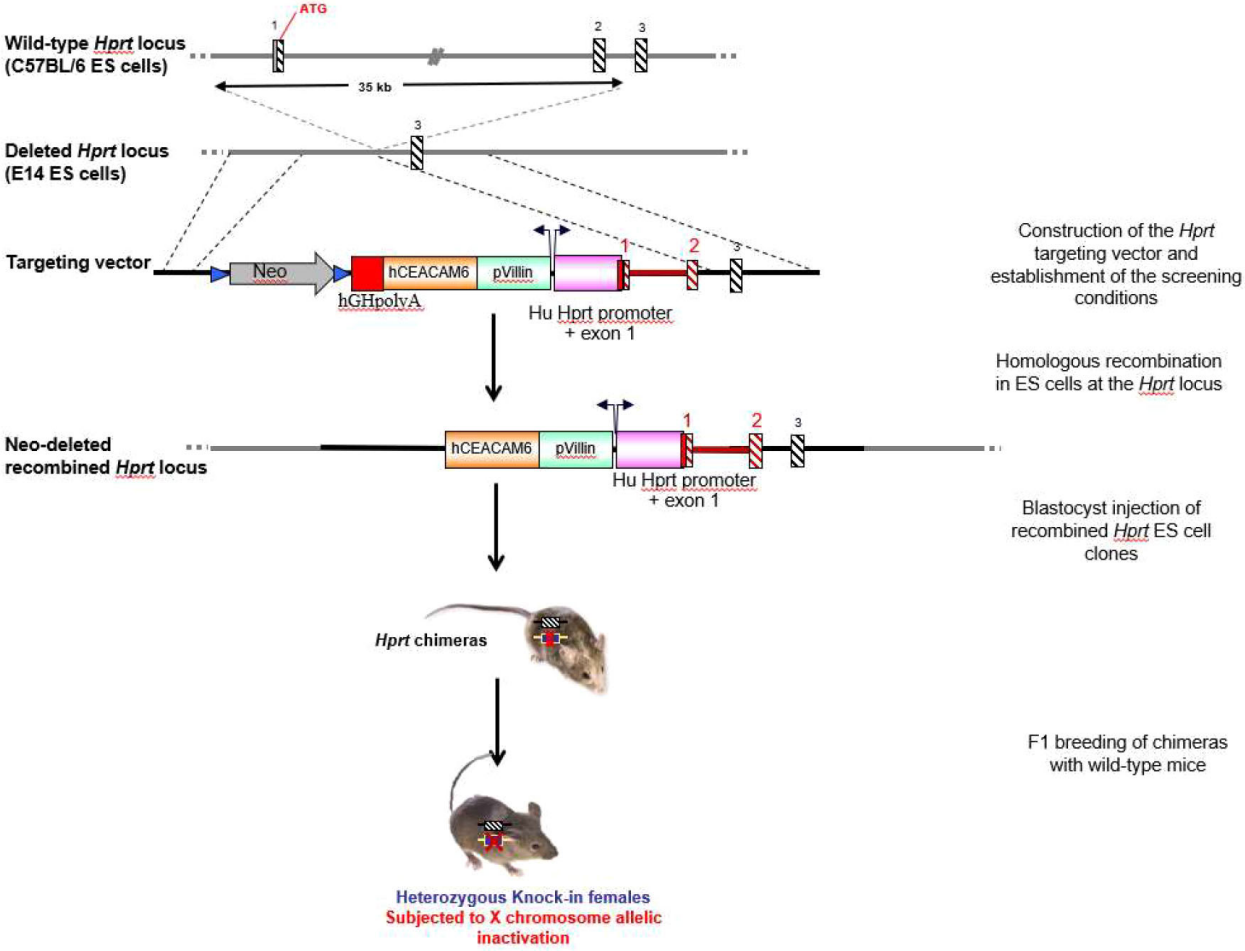
**Generation of a Vill-*h*CC6 knock-in mouse model harbouring the human *CEACAM6* gene under the control of the villin promoter at the *Hprt* locus.** The solid lines represent intronic sequences. In the targeting vector, hatched black and red boxes represent murine and human *Hprt* exons, respectively. Neomycin and loxP sites are represented by a grey arrow and blue triangles, respectively, and the red box depicts hGHpolyA. In recombined E14Tg2a (E14) embryo-derived stem (ES) cells, the *Hprt* gene was reconstituted by introduction of the human promoter (pink box) and exons 1 and 2. Orange and green boxes represent human *CEACAM6* cDNA and the villin promoter, respectively. Recombined ES cell clones were injected into blastocysts to generate chimeric animals. Heterozygous females were obtained following the breeding of the chimeras with wild-type (WT) females. Yellow boxes with red crosses and hatched black boxes represent the knock-in allele and the WT allele, respectively.

**Fig. 2.**
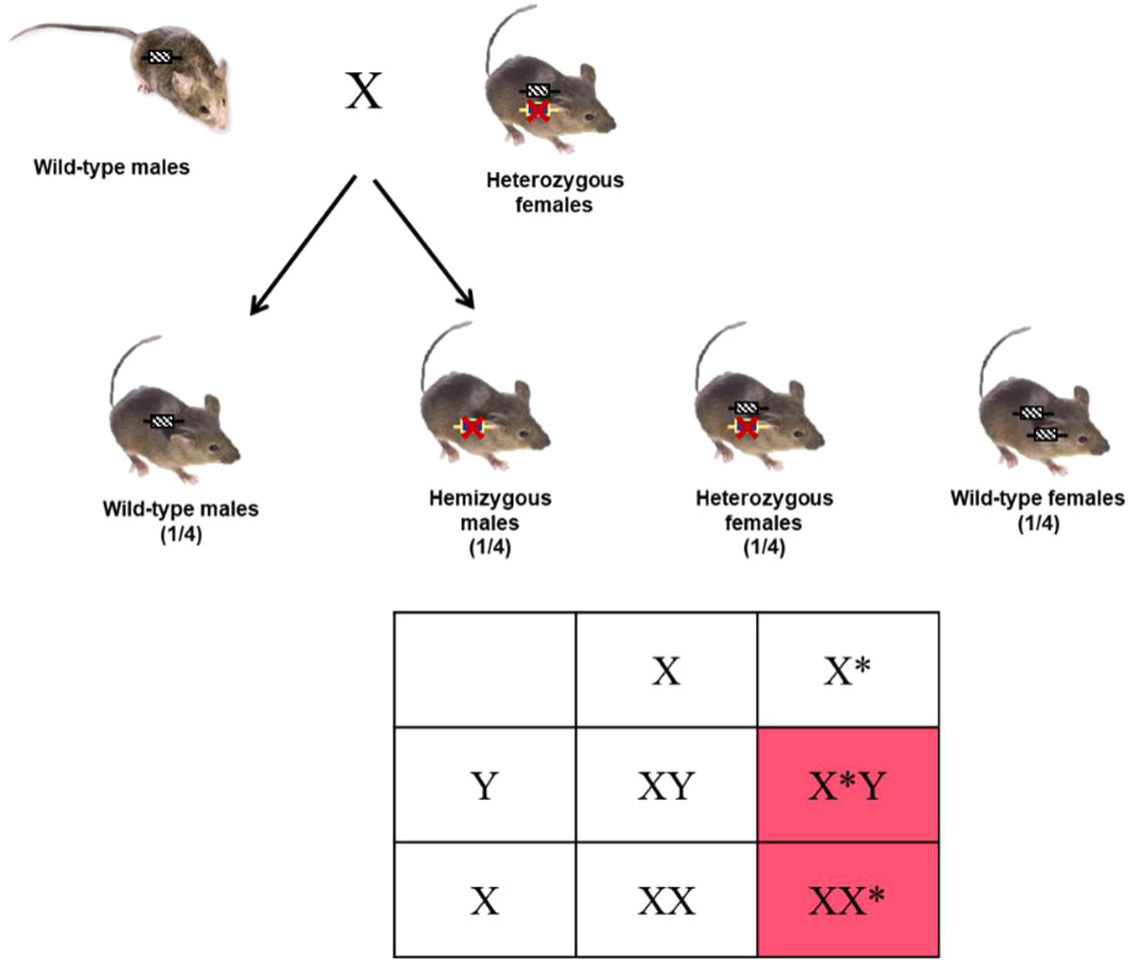
**Global strategy for the generation of hemizygous male mice.** Yellow boxes with red crosses and hatched black boxes represent the knock-in allele and the WT allele, respectively. The asterisks represent the X chromosome carrying the human *CEACAM6* transgene under the control of the villin promoter.

A novel model of transgenic mice expressing the human CEACAM6 protein in the ileal mucosa was generated as described in Fig. 1. The human *CEACAM6* transgene under the control of the mouse villin gene promoter was introduced into the murine *Hprt* locus to generate the knock-in mouse model ‘Vill-*h*CC6’. Heterozygous female mice are viable, fertile, reproduce normally and can nurse their litter naturally. Hemizygous males were obtained by breeding heterozygous females with wild-type (WT) C57BL/6 males (Fig. 2). Knock-in mice do not show evident morphological and developmental alterations or signs of abnormal behaviour.

**Fig. 3.**
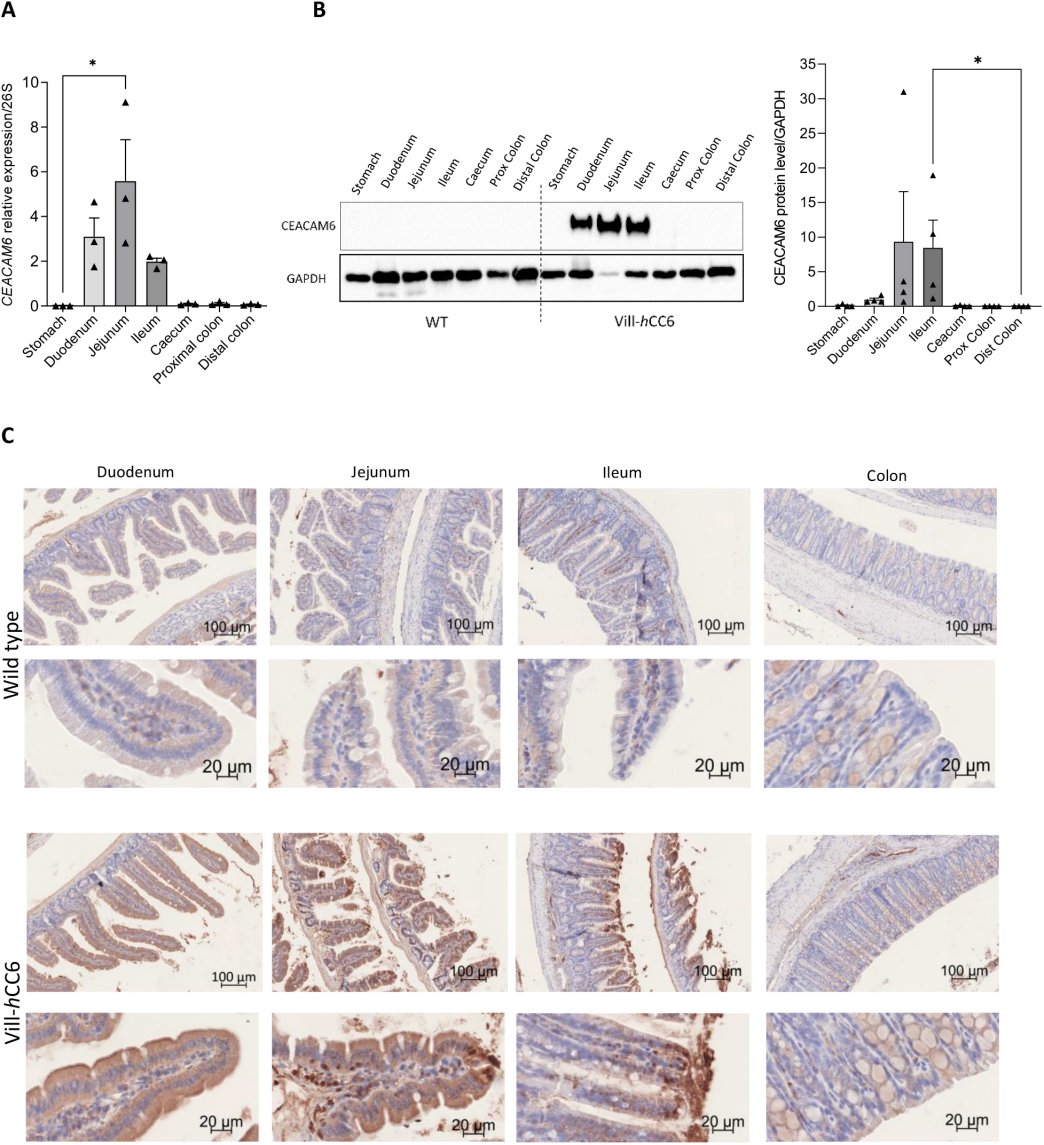
**Mucosal *CEACAM6* expression in the intestine of Vill-*h*CC6 mice.** (A) Segments of the gastrointestinal tract from Vill-*h*CC6 mice were opened and washed with cold PBS, and mucosa were collected by scraping with a glass slide. Total RNA was extracted and used for mRNA analysis. The graph shows the RT-qPCR analysis for mRNA expression levels of *CEACAM6* relative to *Rps26* (26S), *n*=3 mice. Data are presented as mean±s.e.m., *P<0.05 (Kruskal–Wallis test). (B) Total proteins were extracted from mucosa collected from the gastrointestinal tract of WT or Vill-*h*CC6 mice. CEACAM6 accumulation was assessed by western blotting using a monoclonal antibody raised against CEACAM6. Quantification of CEACAM6 expression relative to GAPDH was performed for Vill-*h*CC6 mice by assessing band intensities (*n*=4 mice) using the Image Lab software. Data are presented as mean±s.e.m., *P<0.05 (Kruskal–Wallis test). (C) CEACAM6 immunohistochemical staining of intestinal sections of WT or Vill-*h*CC6 mice. Slides were scanned and assessed with an AxioScan Z1 using Zen 2.3 Pro software.

Mucosal *CEACAM6* expression at different localizations of the intestine of Vill-*h*CC6 hemizygous males was assessed by reverse transcription quantitative polymerase chain reaction (RT-qPCR) (Fig. 3A) and western blotting (Fig. 3B; Fig. S4). As expected, the expression of *CEACAM6*, which is under the control of the villin promoter, is high in the small intestine of Vill-*h*CC6 mice, from the duodenum to the ileum. The CEACAM6 pattern of expression along the gastrointestinal tract was evaluated by immunohistochemical analyses performed on formalin-fixed paraffin-embedded intestinal tissues (Fig. 3C; Fig. S5). The small intestine was divided into three equivalent sections corresponding to the duodenum, jejunum and ileum segments. Strong and specific expression of CEACAM6 was detected in the mucosa of the small intestine of Vill-*h*CC6 mice. Concerning duodenum and jejunum segments, CEACAM6 staining was homogeneous along the villi, and expression was decreased in the crypts. In the ileum, CEACAM6 was concentrated at the apex of the villi, and specific staining appeared in close contact with the epithelium. Expression was faint in the colon of transgenic mice and totally absent in WT mice.

### CEACAM6 overexpression correlates with strong glycosylation at the brush border of enterocytes in Vill-*h*CC6 mice

**Fig. 4.**
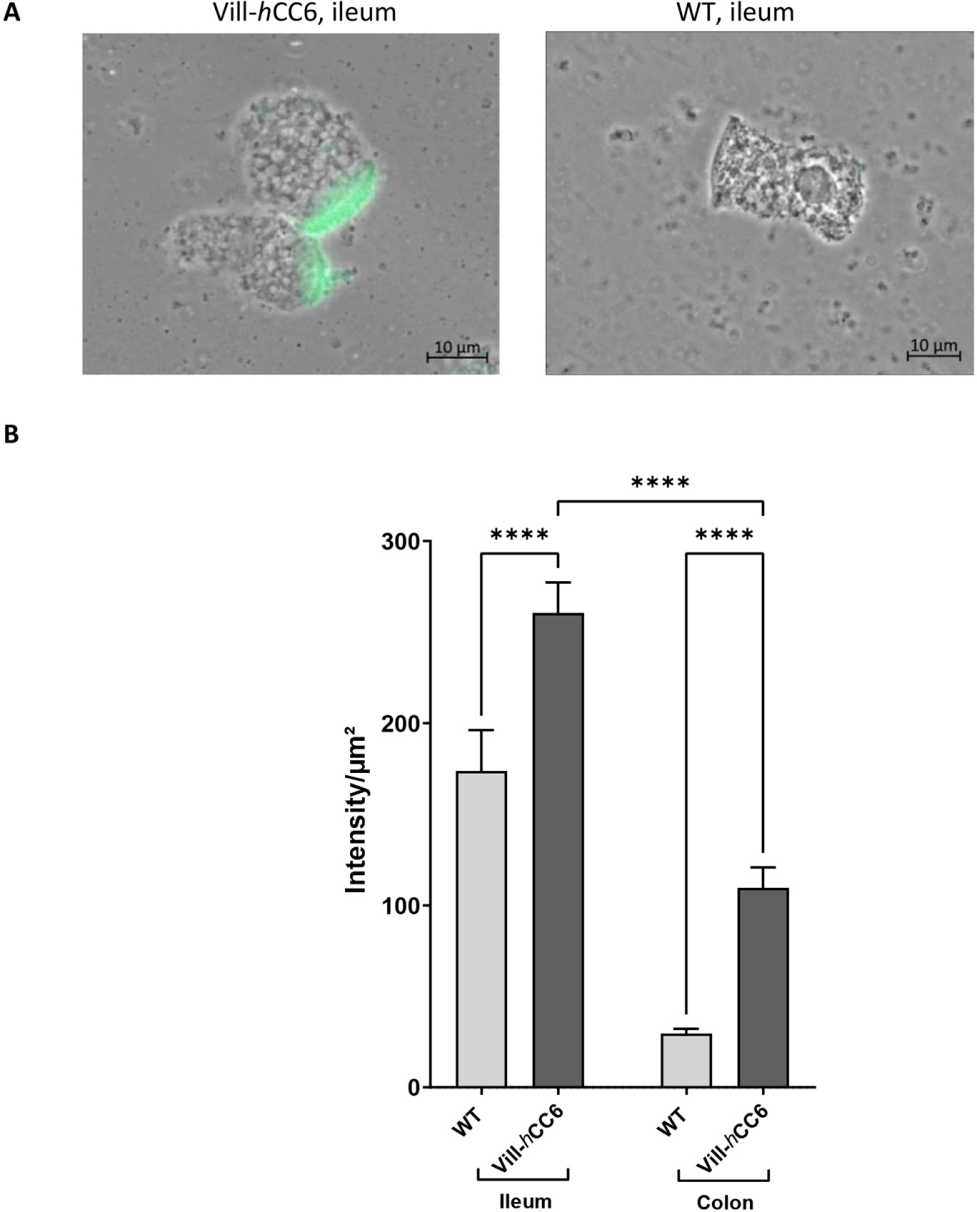
**Analysis of glycosylation at the brush border of enterocytes isolated from intestinal mucosa of Vill-*h*CC6 or WT mice.** Sections (3 cm) of distal ileum or colon were collected from WT or Vill-*h*CC6 mice, and isolated enterocytes were prepared as described in the Materials and Methods section. (A) Enterocytes of WT or Vill-*h*CC6 mice were incubated for 1 h with FITC-labelled concanavalin A at 50 µg/ml in PBS and visualized using phase-contrast microscopy and fluorescence (400×, immersion oil, Axio Observer, Zeiss). (B) The graph shows average fluorescence intensities (intensity/µm^2^) at the brush border of ileal or colonic enterocytes from WT or Vill-*h*CC6 mice (25-30 enterocytes/mouse; three mice/group). Values represent mean±s.e.m., ****P<0.0001 (Kruskal–Wallis test).

In ileal tissues from CD patients, overexpression of *CEACAM6* correlated with high concanavalin A (ConA) lectin staining at the brush border of isolated enterocytes (Barnich et al., 2007). This lectin recognizes d-glucose/d-mannose residues, and one hypothesis is that CEACAM6 may contribute to the high amount of mannose residues exposed at the enterocyte brush border in these patients, which could facilitate AIEC attachment. Fluorescein isothiocyanate (FITC)-labelled ConA staining was performed on isolated enterocytes from the intestine of Vill-*h*CC6 mice and WT littermates. ConA preferentially bound to ileal enterocytes from Vill-*h*CC6 mice compared to WT mice, and fluorescence was focused at the brush border of enterocytes (Fig. 4A). By quantifying the intensity of fluorescence localized at the brush border of enterocytes, we observed that glycosylation was significantly increased in the ileum of Vill-*h*CC6 mice [260.5±16.6 arbitrary units (AU)] compared to WT mice (173.7±22.4 AU, *P*<0.0001), and that difference remained significant for colonic enterocytes (Fig. 4B). Hence, the CEACAM6 expression level in Vill-*h*CC6 mice correlated with the abnormal glycosylation pattern of enterocytes.

### AIEC bacteria preferentially adhere to enterocytes from Vill-*h*CC6 mice in a mannose-dependent manner

**Fig. 5.**
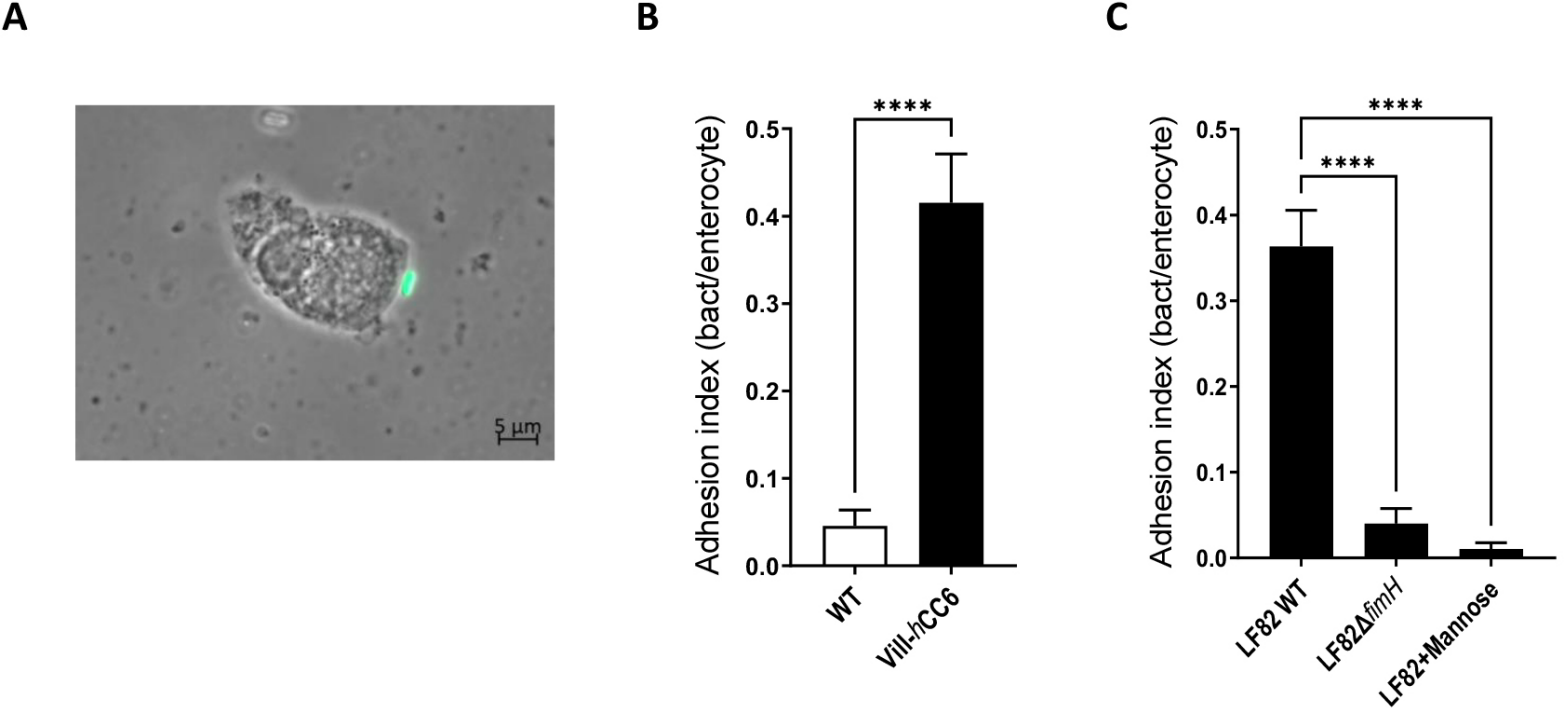
**Assessment of the ability of the adherent-invasive *Escherichia coli* (AIEC) LF82 bacterial strain to adhere to the brush border of ileal enterocytes of Vill-*h*CC6.** Sections (3 cm) of distal ileum were collected from WT or Vill-*h*CC6 mice, and enterocytes were prepared as described in the Materials and Methods section. Enterocytes were infected for 2 h with bacteria (∼10 bacteria/enterocyte) in cell culture medium without antibiotics then were washed in PBS to eliminate nonadherent bacteria. (A) Adhesion of GFP-expressing AIEC LF82 bacteria to the brush border of an ileal enterocyte from a Vill-*h*CC6 mouse (phase-contrast/fluorescence microscopy, 400×, immersion oil, Axio Observer, Zeiss). (B) Adhesion index of GFP-expressing AIEC LF82 bacteria to ileal enterocytes, according to the mouse genotype (total enterocytes counted: 197 for WT mice, 236 for Vill-*h*CC6 mice). (C) Adhesion index to ileal enterocytes from Vill-*h*CC6 mice of a nonpiliated LF82-Δ*fim*H mutant or LF82 WT in the absence or presence of 2% d-mannose (total enterocytes counted: 245 for LF82 WT, 125 for LF82-Δ*fim*H and 192 for LF82+d-mannose). Adhesion index was expressed as the mean number of bacteria adhering to the brush border of one enterocyte±s.e.m. Three independent experiments were performed. ****P<0.0001 (B, Mann–Whitney test; C, Kruskal–Wallis test).

Fresh enterocytes isolated from the ileal mucosa of Vill-*h*CC6 mice or their WT littermates were incubated with GFP-expressing AIEC LF82 bacteria for 2 h. LF82 bacteria firmly adhered to the brush border of ileal enterocytes from Vill-*h*CC6 mice (Fig. 5A). Adhesion ability of bacteria to enterocytes was quantified using an adhesion index, corresponding to the mean number of bacteria adhering to the brush border per enterocyte. Although the adhesion index to ileal enterocytes from WT mice was relatively low (0.046±0.018 bacteria/enterocyte), it was notably and significantly higher in the Vill-*h*CC6 background (0.415±0.056 bacteria/enterocyte, *P*<0.0001) (Fig. 5B). As type 1 pili are the main adhesion factor of the AIEC pathobiont, the ability of an AIEC LF82 mutant deleted for the FimH adhesin-encoding gene (LF82-Δ*fimH*) to adhere to enterocytes was investigated (Fig. 5C). The adhesion index of LF82-Δ*fimH* was drastically decreased compared to that of the WT strain (0.040±0.018 bacteria/enterocyte and 0.363±0.042 bacteria/enterocyte, respectively, *P*<0.0001). Furthermore, the addition of mannose nearly abrogated the bacterial adhesion of the AIEC LF82 WT strain to the brush border (0.010±0.007 bacteria/enterocyte, *P*<0.0001). Thus, CEACAM6 and type 1 pili play a crucial role in the ability of AIEC LF82 to adhere to intestinal mucosa in the Vill-*h*CC6 mouse model.

### AIEC bacteria preferentially persist in the gut of Vill-*h*CC6 mice

**Fig. 6.**
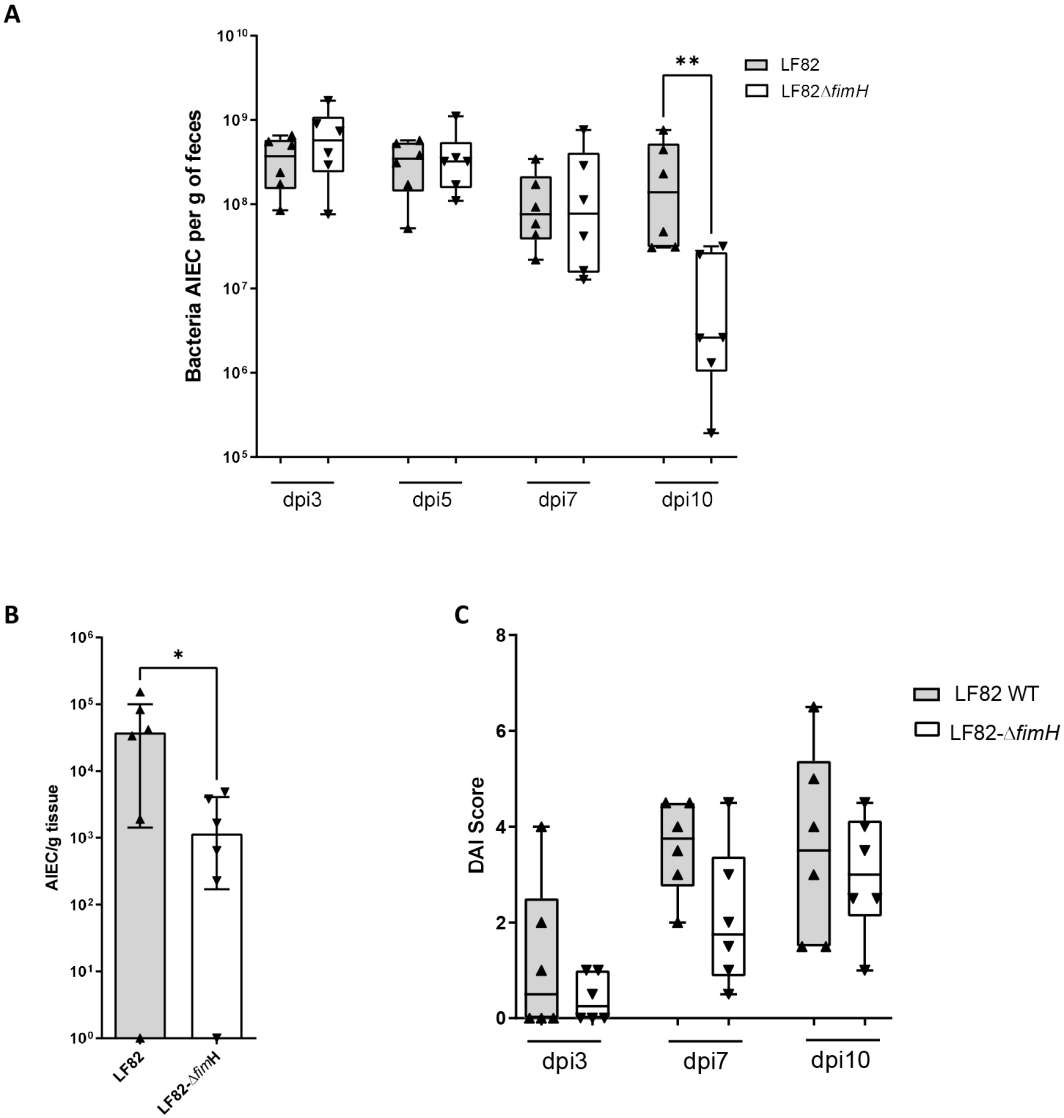
**Ability of the AIEC LF82 WT strain or of the nonpiliated LF82-Δ*fimH* mutant to colonize Vill-*h*CC6 mice.** Vill-*h*CC6 mice were treated with streptomycin for 2 days (day −3) and then received water (day −1) for 24 h. Mice were orally administered (day 0) 10^9^ colony-forming units (CFU) of AIEC LF82 or LF82-Δ*fimH* bacteria and were killed at 10 days postinfection (dpi). (A) Quantification of AIEC bacteria in the faeces at 3, 5, 7 and 10 dpi (CFU/g of faeces). (B) Quantification of AIEC bacteria associated with the ileal mucosa at 10 dpi (CFU/g of tissue). (C) Disease activity index (DAI) score assessment at 3, 7 and 10 dpi. *n*=6 per group. Values represent median with interquartile range, *P<0.05, **P<0.01 (Mann–Whitney test).

The transgenic mouse model Vill-*h*CC6 expressing CEACAM6 in ileal epithelial cells represents an interesting new preclinical model to analyse ileal tropism of AIEC and therefore to assess promising anti-AIEC strategies. To investigate the role of the FimH adhesin in the intestinal encroachment of the AIEC pathobiont, Vill-*h*CC6 mice were challenged with the AIEC LF82 WT strain or with the LF82-Δ*fimH* mutant. Faecal AIEC bacteria, reflecting the gut persistence of the pathobiont, were assessed for 10 days. At 10 days postinfection (10 dpi), the bacterial load was significantly decreased in the faeces of mice receiving the nonpiliated mutant, whereas the levels of WT LF82 bacteria remained high [2.61×10^6^ colony-forming units (CFU)/g and 1.39×10^8^ CFU/g, respectively] (Fig. 6A). Furthermore, the LF82-Δ*fimH* mutant was less able to adhere to the ileal mucosa and induced fewer clinical signs of disease, as reflected by the disease activity index (DAI) score assessment (Fig. 6B,C). In conclusion, gut establishment of the AIEC pathobiont in Vill-*h*CC6 mice involves the bacterial FimH adhesin.

**Fig. 7.**
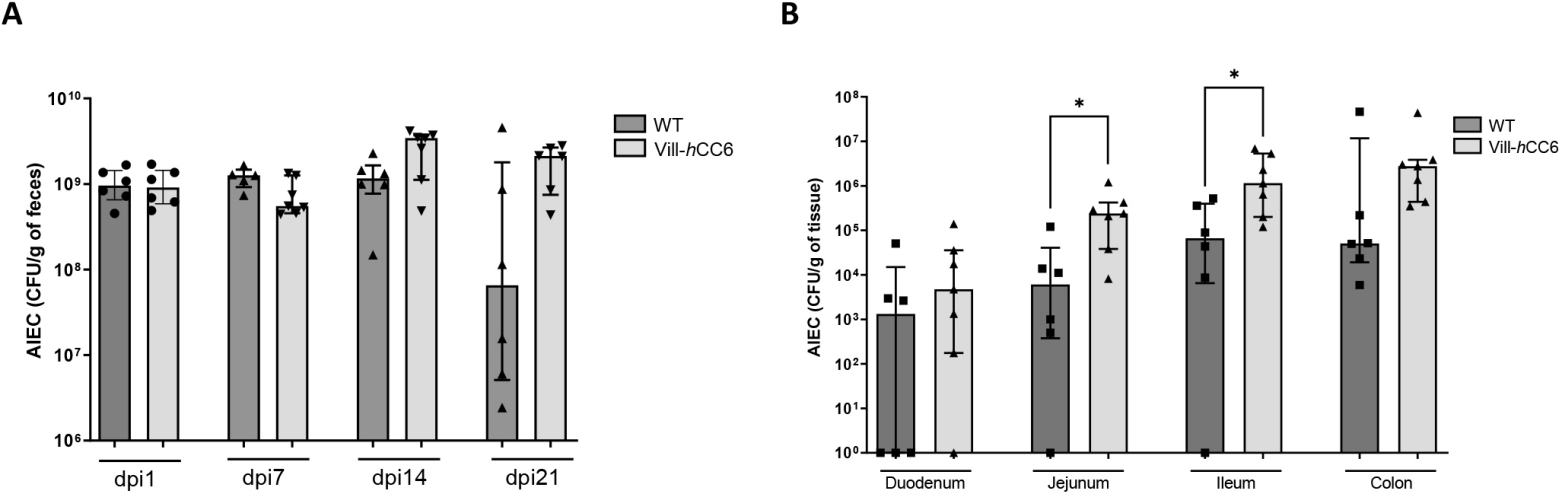
**AIEC gut colonization after an oral challenge with a mix of four AIEC strains (CEA614S, CEA618U, CEA212U and CEA304S) in Vill-*h*CC6 or WT mice.** Vill-*h*CC6 or WT mice were treated with streptomycin for 2 days (day −3) and then received water (day −1) for 24 h. Mice were orally administered (day 0) 10^9^ CFU of AIEC bacterial mix (CEA614S, CEA618U, CEA212U and CEA304S) and were killed at day 21 after administration. (A) Quantification of AIEC bacteria in the faeces at 1, 7, 14 and 21 dpi (CFU/g of faeces). (B) Quantification of AIEC bacteria associated with the intestinal mucosa at 21 dpi (CFU/g of tissue). WT mice, *n*=6; Vill-*h*CC6 mice, *n*=7. Values represent median with interquartile range, *P<0.05 (Mann–Whitney test).

The experiment was then performed with other clinical strains of AIEC recently isolated from ileal biopsies of CD patients. Mice were challenged with a mix of four AIEC strains (CEA614S, CEA618U, CEA212U, CEA304S). Bacteria were quantified in the faeces for 21 days as the bacterial load was still high in the faeces of both groups at 14 dpi, and mucosa-associated bacteria were numbered at euthanasia. Although the AIEC bacterial load remained high and stable for 21 days in the Vill-*h*CC6 mice (2.12×10^9^ CFU/g of faeces at 21 dpi), it decreased in WT mice at 21 dpi (6.53×10^7^ CFU/g of faeces) (Fig. 7A). Moreover, significantly more AIEC bacteria were associated with the jejunal and ileal mucosa of transgenic mice compared to those of WT mice (Fig. 7B). Taken together, these data show that AIEC gut colonization is favoured in the Vill-*h*CC6 transgenic model.

## DISCUSSION

Future investigations of AIEC pathogenicity and of suitable therapeutic strategies targeting this pathobiont depend on robust animal models and preclinical experimental data. Previous studies demonstrated the key role of CEACAM6 in the ability of AIEC bacteria to colonize intestinal mucosa. Although CEACAM6 is highly present in the human colonic mucosa, the expression of this glycoprotein is very low and even absent in the ileal segment. However, in more than 35% of CD patients, abnormal expression of CEACAM6 is observed, and this overexpression favours AIEC attachment to the epithelium (Barnich et al., 2007). A transgenic mouse model named CEABAC10 and carrying several human CEACAM genes (integrated into a 187-kb bacterial artificial chromosome, among which *CEACAM6*) is already available (Chan and Stanners, 2004). CEABAC10 mice allowed us to better appreciate the role of AIEC bacteria in inflammatory bowel diseases by demonstrating the involvement of the CEACAM6 glycoprotein in the ability of AIEC strains to adhere to the intestinal epithelium (Carvalho et al., 2009), and to better understand how this colonization modulated intestinal permeability, in particular by inducing the expression of pore-forming claudin 2 (Denizot et al., 2012). They also enabled us to reveal how environmental factors, such as a Western-style diet, favour the gut encroachment of AIEC bacteria (Martinez-Medina et al., 2014; Agus et al., 2014). Furthermore, this model has highlighted in preclinical studies the efficacy of various anti-AIEC strategies, i.e. phage cocktail (EcoActive), various synthetic molecules based on mannose to block the interaction of FimH with CEACAM6, or yeast probiotics such as *Saccharomyces cerevisiae* (whole yeast or yeast extracts) to prevent the implantation of, or to eliminate, AIEC bacteria from the gut (Galtier et al., 2017; Sivignon et al., 2015a,b, 2021). However, the limitation of this model relies on the fact that CEACAM6 expression is mainly located in the colon, whereas it remains very low in the small intestine. Thus, the mechanisms of AIEC–intestinal epithelium interactions as well as the ability of anti-AIEC strategies to limit/eliminate bacteria have only been investigated at the colon level in this model.

In gnotobiotic mice monocolonized with AIEC bacteria, the results showed that AIEC bacteria require flagellar mobility to cross the mucus layer and to reach the surface of the epithelium (Sevrin et al., 2020). Various mouse models also highlighted that mucosal metabolites support the general growth of AIEC in the gut, with a selective effect of ethanolamine or serine on AIEC blooming (Zhang et al., 2022; Kitamoto et al., 2020). Another work performed in mice with simplified microbiota (altered Schaedler flora) showed that the addition of AIEC to this ecosystem disrupted microbiota composition and increased levels of bioactive lipopolysaccharide and flagellin, but only modestly promoted some parameters of intestinal inflammation (Chassaing and Gewirtz, 2018). Moreover, genetically susceptible mice with normal microbiota, such as *Eif2ak4*^−/−^ mice, exhibiting autophagy defects in response to AIEC infection, demonstrated that AIEC implantation can induce chronic intestinal inflammation via modulation of the composition of the gut microbiota (Bretin et al., 2018). Finally, mouse models in which intestinal inflammation is chemically induced (DSS) or induced by environmental factors (such as a high-fat diet or dietary emulsifiers) indicated that AIEC bacteria can aggravate a pre-existing inflammatory state at the intestinal level (Carvalho et al., 2008; Martinez-Medina et al., 2014; Viennois et al., 2020). Although these models have driven progress in understanding the role of AIEC bacteria in the pathophysiology of inflammatory bowel disease, they do not mimic the ileal AIEC colonization observed in CD patients with ileal involvement.

This limitation led us to develop a new animal model. The original work of our research group showed that AIEC strains were found only in colonic specimens of 3.7% of CD patients versus 1.9% of controls (Darfeuille-Michaud et al., 2004). However, AIEC are especially associated with the ileal mucosa. Indeed, AIEC were found in 21.7% of CD chronic ileal lesions versus in 6.2% of controls, and, in neoterminal ileal specimens, they were found in 36.4% of CD early lesions. Since then, these results have been confirmed by other teams around the world (Kamali Dolatabadi et al., 2021; Nadalian et al., 2021; Palmela et al., 2018). More recently, we investigated the role of AIEC bacteria in CD, taking advantage of a large prospective cohort of patients with CD undergoing ileocolonic resection. We observed a twofold higher prevalence of AIEC bacteria within biopsies from the neoterminal ileum at 6 months postsurgery compared with surgical specimens (ileal side); moreover, ileal colonization was associated with the early phase of ileal CD recurrence (Buisson et al., 2022).

Thus, to deeply investigate the pathogenicity of the AIEC pathobiont in relation to its attachment to CEACAM6, a novel mouse model was generated, named ‘Vill-*h*CC6’, with strong CEACAM6 expression in the ileal mucosa. Interestingly, AIEC bacteria were able to highly persist in the gut of mice for up to 21 days after a unique infection, with a significant higher quantity of bacteria associated with jejunal and ileal mucosa in transgenic mice expressing CEACAM6 compared to WT mice. Bacterial attachment to mucosa is associated with a high glycosylation state of CEACAM6-expressing epithelial cells that could thus be targeted by the FimH bacterial adhesin. Indeed, an AIEC mutant deleted from *fimH* was less able to adhere to the ileal mucosa of Vill-*h*CC6 mice. These data clearly support our hypothesis that CEACAM6 favours AIEC attachment to small intestine mucosa and that FimH plays a crucial role in this interaction. Thus, AIEC-infected Vill-*h*CC6 mice, modelling ileal CD patients colonized by AIEC pathobionts, could be used in the future as a more appropriate animal model to evaluate, over one or more generations, preventive strategies to limit AIEC ileal colonization, such as the use of natural extracts incorporated in the diet or the use of various probiotics and/or protective microorganisms. Furthermore, the efficacy of new strategies to eliminate AIEC bacteria associated with the ileal mucosa will also be tested in this model, such as phage treatment, inhibitory and/or antivirulence molecules, faecal microbiota transplantation or, as an example, CRISPR-based strategies targeting virulence or fitness factors of AIEC bacteria.

## MATERIALS AND METHODS

### Generation of the Vill-*h*CC6 knock-in mouse model

Vill-*h*CC6 knock-in mice were generated by genOway (Lyon, France). The human *CEACAM6* cDNA (NM_002483) under the control of the villin promoter was introduced into the hypoxanthine phosphoribosyltransferase (*Hprt*) locus by homologous recombination (Fig. 1). The knock-in mice were developed using E14Tg2a [embryonic day (E)14] embryo-derived stem (ES) cells from the 129P2/OlaHsd mouse strain. The targeted insertion repairs the *Hprt* gene deletion in E14 ES cells and rescues the expression of the endogenous *Hprt* gene. After transfection, E14 ES cells with a functional *Hprt* gene were selected using neomycin positive selection (a neomycin cassette flanked by loxP sites was added to the targeting vector) and HAT medium containing hypoxanthine, aminopterin and thymidine substrates to enrich for ES cell clones showing the correct targeting event. Southern blot analyses were performed to confirm the presence of the recombined allele (Fig. S1). ES cell clones were then injected into C57BL/6 blastocysts and implanted into OF-1 pseudopregnant females. Highly chimeric males were selected for breeding with WT C57BL/6 Cre Deleter females to excise the neomycin selection cassette and to generate heterozygous females carrying the neo-excised knock-in allele. This resulting F1 generation of heterozygous mice was genotyped by PCR to confirm the presence of the transgene (conditions and results are detailed in Table S1 and Fig. S2).

Because the *Hprt* knock-in locus is localized on the X chromosome, it is preferable to analyse the expression of the *CEACAM6* transgene in males (hemizygous) or homozygous females to avoid dealing with the allelic inactivation described for the X chromosome (Lyon, 1961). Hemizygous males were produced from the second generation F2 by breeding WT C57BL/6 males with PCR-validated heterozygous females (Fig. 2). The integrity of the *Hprt* knock-in locus containing the *CEACAM6* gene was further verified by DNA sequencing (Fig. S3). The Vill-*h*CC6 mouse model was crossed in specific pathogen-free conditions at least nine times with the C57BL/6 strain (Charles River Laboratories, France) to ensure a complete C57BL/6 genetic background and then to maintain the established transgenic model.

### Animals and ethics

This study was carried out in strict accordance with the recommendations of the Guide for the Care and Use of Laboratory Animals of Clermont Auvergne University (Clermont-Ferrand, France). The animal protocol was approved by the Committee for Research and Ethical Issues of the Department of Auvergne (CEMEA Auvergne; Permit Number: CEMEAA, 2018031914539228).

Mice were housed in a temperature-controlled room (21-22°C) with a 12:12-h light–dark cycle in individually ventilated polysulfone cages with an internal area of 500 cm^2^ and 18.0 cm depth, wood bedding and appropriate enrichments. Up to a maximum of five animals were housed per cage, with access to food and water *ad libitum*, in the animal facility of Clermont Auvergne University.

### Bacterial strains and culture conditions

The AIEC strains LF82, CEA614S, CEA618U, CEA212U and CEA304S used in this study were isolated from the ileal mucosa of CD patients requiring ileocolonoscopy (Darfeuille-Michaud et al., 2004; Buisson et al., 2021). AIEC LF82 is naturally resistant to ampicillin and erythromycin. For CEA614S, CEA618U, CEA212U and CEA304S, streptomycin-resistant mutants (AIEC-Stp^R^) were constructed by insertion of the streptomycin resistance-encoding gene on the chromosome using a mobilizable mini-Tn7-based vector (pUC18R6KT-mini-Tn7T-km-*Stp*). Mini-Tn7 delivery was accomplished by three parental matings involving *E. coli* MFDpir46 harbouring the respective mini-Tn7 delivery plasmid, the AIEC recipient strain, and *E. coli* MFDpir/pTNS3 encoding the *tnsABCD* genes necessary for the transposition of mini-Tn7 at the *attTn7* insertion site (Ferrières et al., 2010). The LF82-GFP strain was constructed using this method and the pUC18R6KT-mini-Tn7T-km-*GFP* plasmid. A nonpiliated chromosomal mutant, LF82-Δ*fimH*, was used as a control (Dreux et al., 2013). The bacterial strains were grown in Luria Bertani (LB) broth under static and aerobic conditions overnight at 37°C. For *in vivo* experiments, overnight bacterial cultures of AIEC were harvested by centrifugation at 4500 ***g*** for 10 min. Bacterial pellets were resuspended in PBS to reach a concentration of 5×10^9^ bacteria/ml.

### RNA extraction and RT-qPCR

Total RNA from the mucosa of different sections of the intestine was extracted using an RNeasy Mini Kit (Qiagen, France) following the manufacturer's instructions. RNA quality was assessed by a NanoPhotometer^®^ (Implen, Germany). Reverse transcription was performed on 500 ng RNA using a PrimeScript™ RT Reagent Kit (TaKaRa, France). cDNA was diluted (1/10), and 3 µl was used for qPCR quantification (iTaq Universal SYBR Green Supermix, Bio-Rad, France). *CEACAM6* relative expression compared to that of the housekeeping gene *Rps26* (ribosomal protein S26, mRNA) was determined using the 2^−ΔCt^ method. The primer sequences used were as follows: *CEACAM6* Forward, 5′-GAATGAAGAAGCAACCGGAC-3′; *CEACAM6* Reverse, 5′-CAGGTAGGTTGTGTTCTGAAC-3′; *Rps26* Forward, 5′-TGTCATTCGGAACATTGTAG-3′; *Rps26* Reverse, 5′-GGCTTTGGTGGAGGTC-3′.

### Protein extraction and western blotting

Mucosa from different sections of the gastrointestinal tract (stomach, duodenum, jejunum, ileum, caecum and colon) of Vill-*h*CC6 and WT mice were scraped with a glass slide and suspended in 300 µl lysis buffer (25 mM Tris-HCl pH 7.5, 1 mM EDTA, 5 mM EGTA pH 8.0, 1 mM MgCl_2_, 1% NP-40, 10% glycerol and 150 mM NaCl). After vortexing, the samples were incubated for 10 min on ice, disrupted for 10 min and centrifuged at 19,000 ***g*** for 10 min at 4°C. Supernatants were collected, and total protein contents were assayed with a DC Protein Assay (Bio-Rad). Laemmli buffer was added to the protein extracts (1:4) before boiling (5 min at 99°C). Protein extracts were loaded in a 12% SDS-PAGE gel for electrophoresis migration before transfer to a nitrocellulose membrane (Trans-Blot^®^ Turbo™ Transfer System, Bio-Rad). Membranes were incubated for 1 h in blocking buffer (PBS-0.05% Tween 20, 5% bovine serum albumin) and then blotted with the primary antibodies anti-CEACAM6 (1:5000, 9A6 clone, Genovac GmbH, Germany) and anti-GAPDH (1:5000, Cell Signaling Technology, USA) overnight at 4°C. After three washes with PBS-0.05% Tween 20, the membranes were incubated for 1 h at room temperature (RT) with horseradish peroxidase-conjugated secondary antibodies diluted in blocking buffer (1:10,000). Membranes were washed three times, and proteins were detected using Enhanced Chemiluminescence substrate (Clarity™ Western ECL substrate, Bio-Rad). Quantification of CEACAM6 expression relative to GAPDH was performed for Vill-*h*CC6 mice by assessing band intensities (*n*=4 mice) using Image Lab software 6.1.0 (Bio-Rad Laboratories Inc.)

### Analysis of CEACAM6 expression by immunohistochemistry

Duodenum, jejunum, ileum or colon samples from WT or Vill-*h*CC6 mice were fixed in formalin solution (neutral buffered, 10%, Sigma-Aldrich, France) and embedded in paraffin. Slides of intestinal tissues (5 µm) underwent unwaxing, rehydration, and antigen retrieval with citrate buffer, and were incubated with 0.3% hydrogen peroxide (H_2_O_2_) for 30 min to inactivate endogenous peroxidase activity. The sections were blocked using PBS with 1.5% normal goat serum for 1 h at RT and incubated with anti-CEACAM6 antibody (1:500, 9A6 clone, GM-0509, Genovac GmbH) diluted in PBS with goat serum 1.5% overnight at 4°C. Then, tissues were incubated with an anti-mouse biotinylated secondary antibody (1:500, Interchim, France) for 1 h at RT before incubation with peroxidase-conjugated streptavidin (Jackson ImmunoResearch, UK) for 30 min at RT. For visualization, a Vector^®^ NovaRED^®^ Substrate Kit (Vector Laboratories, USA) was used, and tissues were counterstained with Mayer's Haematoxylin. Tissues were then dehydrated, and slides were mounted using Cytoseal™ 60. Slides were scanned with an AxioScan Z1 (Zeiss) and analysed using Zen 2.3 Pro software.

### ConA staining and infection experiments on isolated enterocytes from mice

Enterocytes were isolated from ileal and colonic sections from WT or Vill-*h*CC6 mice. Briefly, intestinal sections were longitudinally opened and washed in PBS, and mucosa were scraped with a glass slide. Mucosal suspensions were washed three times in PBS and then incubated in Hanks' balanced salt solution (HBSS)-EDTA 2 mM (Merck, Germany) for 15 min with orbital shaking. Enterocytes were then incubated for 1 h at RT with FITC-labelled ConA (Vector Laboratories, USA) at 50 µg/ml in PBS. After two successive washes, enterocytes were visualized using phase-contrast microscopy and brush borders areas were manually bounded. Glycosylation levels at the brush border of enterocytes were assessed by measuring the average fluorescence intensities (intensity/µm^2^) in brush border areas using Zen Pro 3.3 software (Axio Observer, Zeiss, France).

For AIEC infection assays, fresh enterocytes isolated from the ileum of WT or Vill-*h*CC6 mice were prepared as previously described. Enterocytes (∼4×10^6^ cells) were infected for 2 h at RT with 4×10^7^ AIEC bacteria in minimum essential medium supplemented with 10% heat-inactivated foetal calf serum in the presence or absence of 2% d-mannose (Merck, Germany). Nonadherent bacteria were eliminated with two washes with PBS. Adhering bacteria were visualized using phase-contrast/fluorescence microscopy. The adhesion index was determined by counting the mean number of AIEC bacteria associated with the brush border of enterocytes using phase-contrast microscopy (Axio Observer, Zeiss).

### Mouse infection protocol

Vill-*h*CC6 or WT male mice (6-8 weeks old) were orally pretreated for 2 days with streptomycin (2.5 g/l) in drinking water to disrupt normal resident (bacterial) intestinal microbiota. Twenty-four hours later, the mice were challenged with 10^9^ AIEC bacteria (in 0.2 ml of PBS). Body weight was monitored throughout the experiment. Fresh faecal pellets (≈100 mg) were collected at different dpi and homogenized in PBS. Bacteria were numbered by plating appropriate dilutions on LB agar medium containing the appropriate antibiotics to select and isolate AIEC bacteria (LF82 WT and LF82-Δ*fimH*, 100 µg/ml ampicillin/20 µg/ml erythromycin; AIEC-Stp^R^ strains, 100 µg/ml streptomycin) and incubated overnight at 37°C. The severity of colitis was assessed by the DAI score, which ranges from 0 (healthy) to 12 (high colitis activity) as previously described (Sivignon et al., 2015b; Table S2). At 10 or 21 dpi, the mice were anesthetized with isoflurane and killed by cervical dislocation. Intestinal tissues were collected, washed and homogenized in PBS before plating onto antibiotic-selective culture media to count AIEC bacteria associated with the tissue.

### Statistical analysis

Values are expressed as the mean±s.e.m. or as the median. Statistical analyses were performed using Prism 9 (version 9.3.1, GraphPad Software, USA). An unpaired Mann–Whitney test was performed for single comparisons, and the Kruskal–Wallis test was performed for multiple comparisons. *P*<0.05 was considered statistically significant.

## Supplementary Material

10.1242/dmm.049707_sup1 Supplementary information

## References

[DMM049707C1] Agus, A., Massier, S., Darfeuille-Michaud, A., Billard, E. and Barnich, N. (2014). Understanding host-adherent-invasive *Escherichia coli* interaction in Crohn's disease: opening up new therapeutic strategies. *Biomed. Res. Int.* 2014, 567929. 10.1155/2014/567929 25580435 PMC4279263

[DMM049707C2] Barnich, N., Carvalho, F. A., Glasser, A.-L., Darcha, C., Jantscheff, P., Allez, M., Peeters, H., Bommelaer, G., Desreumaux, P., Colombel, J.-F. et al. (2007). CEACAM6 acts as a receptor for adherent-invasive *E. coli*, supporting ileal mucosa colonization in Crohn disease. *J. Clin. Invest.* 117, 1566-1574. 10.1172/JCI30504 17525800 PMC1868786

[DMM049707C3] Boudeau, J., Barnich, N. and Darfeuille-Michaud, A. (2001). Type 1 pili-mediated adherence of *Escherichia coli* strain LF82 isolated from Crohn's disease is involved in bacterial invasion of intestinal epithelial cells. *Mol. Microbiol.* 39, 1272-1284. 10.1111/j.1365-2958.2001.02315.x 11251843

[DMM049707C4] Bretin, A., Lucas, C., Larabi, A., Dalmasso, G., Billard, E., Barnich, N., Bonnet, R. and Nguyen, H. T. T. (2018). AIEC infection triggers modification of gut microbiota composition in genetically predisposed mice, contributing to intestinal inflammation. *Sci. Rep.* 8, 12301. 10.1038/s41598-018-30055-y 30120269 PMC6098085

[DMM049707C5] Buisson, A., Vazeille, E., Fumery, M., Pariente, B., Nancey, S., Seksik, P., Peyrin-Biroulet, L., Allez, M., Ballet, N., Filippi, J. et al. (2021). Faster and less invasive tools to identify patients with ileal colonization by adherent-invasive *E. coli i*n Crohn's disease. *United European Gastroenterol. J.* 9, 1007-1018. 10.1002/ueg2.12161 PMC8598958 34791806

[DMM049707C6] Buisson, A., Sokol, H., Hammoudi, N., Nancey, S., Treton, X., Nachury, M., Fumery, M., Hébuterne, X., Rodrigues, M., Hugot, J.-P. et al. (2022). Role of adherent and invasive *Escherichia coli* in Crohn's disease: lessons from the postoperative recurrence model. *Gut* gutjnl-2021-325971. 10.1136/gutjnl-2021-325971 35361684

[DMM049707C7] Carvalho, F. A., Barnich, N., Sauvanet, P., Darcha, C., Gelot, A. and Darfeuille-Michaud, A. (2008). Crohn's disease-associated *Escherichia coli* LF82 aggravates colitis in injured mouse colon *via* signaling by flagellin. *Inflamm. Bowel Dis.* 14, 1051-1060. 10.1002/ibd.20423 18338780

[DMM049707C8] Carvalho, F. A., Barnich, N., Sivignon, A., Darcha, C., Chan, C. H. F., Stanners, C. P. and Darfeuille-Michaud, A. (2009). Crohn's disease adherent-invasive *Escherichia coli* colonize and induce strong gut inflammation in transgenic mice expressing human CEACAM. *J. Exp. Med.* 206, 2179-2189. 10.1084/jem.20090741 19737864 PMC2757893

[DMM049707C9] Chan, C. H. F. and Stanners, C. P. (2004). Novel mouse model for carcinoembryonic antigen-based therapy. *Mol. Ther.* 9, 775-785. 10.1016/j.ymthe.2004.03.009 15194045

[DMM049707C10] Chassaing, B. and Gewirtz, A. T. (2018). Mice harboring pathobiont-free microbiota do not develop intestinal inflammation that normally results from an innate immune deficiency. *PLoS ONE* 13, e0195310. 10.1371/journal.pone.0195310 29617463 PMC5884553

[DMM049707C11] Chervy, M., Barnich, N. and Denizot, J. (2020). Adherent-invasive *E. coli*: update on the lifestyle of a troublemaker in Crohn's disease. *Int. J. Mol. Sci.* 21, 3734. 10.3390/ijms21103734 PMC7279240 32466328

[DMM049707C12] Darfeuille-Michaud, A., Boudeau, J., Bulois, P., Neut, C., Glasser, A.-L., Barnich, N., Bringer, M.-A., Swidsinski, A., Beaugerie, L. and Colombel, J.-F. (2004). High prevalence of adherent-invasive *Escherichia coli* associated with ileal mucosa in Crohn's disease. *Gastroenterology* 127, 412-421. 10.1053/j.gastro.2004.04.061 15300573

[DMM049707C13] Denizot, J., Sivignon, A., Barreau, F., Darcha, C., Chan, C. H. F., Stanners, C. P., Hofman, P., Darfeuille-Michaud, A. and Barnich, N. (2012). Adherent-invasive *Escherichia coli i*nduce claudin-2 expression and barrier defect in CEABAC10 mice and Crohn's disease patients. *Inflamm. Bowel Dis.* 18, 294-304. 10.1002/ibd.21787 21688348

[DMM049707C14] Douadi, C., Vazeille, E., Chambon, C., Hébraud, M., Fargeas, M., Dodel, M., Coban, D., Pereira, B., Birer, A., Sauvanet, P. et al. (2022). Anti-TNF agents restrict adherent-invasive *Escherichia coli* replication within macrophages through modulation of chitinase 3-like 1 in patients with Crohn's disease. *J. Crohns Colitis* 16, 1140-1150. 10.1093/ecco-jcc/jjab236 35022663

[DMM049707C15] Dreux, N., Denizot, J., Martinez-Medina, M., Mellmann, A., Billig, M., Kisiela, D., Chattopadhyay, S., Sokurenko, E., Neut, C., Gower-Rousseau, C. et al. (2013). Point mutations in FimH adhesin of Crohn's disease-associated adherent-invasive *Escherichia coli* enhance intestinal inflammatory response. *PLoS Pathog.* 9, e1003141. 10.1371/journal.ppat.1003141 23358328 PMC3554634

[DMM049707C16] Ferrières, L., Hémery, G., Nham, T., Guérout, A.-M., Mazel, D., Beloin, C. and Ghigo, J.-M. (2010). Silent mischief: bacteriophage Mu insertions contaminate products of *Escherichia coli* random mutagenesis performed using suicidal transposon delivery plasmids mobilized by broad-host-range RP4 conjugative machinery. *J. Bacteriol.* 192, 6418-6427. 10.1128/JB.00621-10 20935093 PMC3008518

[DMM049707C17] Galtier, M., De Sordi, L., Sivignon, A., de Vallée, A., Maura, D., Neut, C., Rahmouni, O., Wannerberger, K., Darfeuille-Michaud, A., Desreumaux, P. et al. (2017). Bacteriophages targeting adherent invasive *Escherichia coli* strains as a promising new treatment for Crohn's disease. *J. Crohns Colitis* 11, 840-847. 10.1093/ecco-jcc/jjw224 28130329

[DMM049707C18] Kamali Dolatabadi, R., Feizi, A., Halaji, M., Fazeli, H. and Adibi, P. (2021). The prevalence of adherent-invasive Escherichia coli and its association with inflammatory bowel diseases: a systematic review and meta-analysis. *Front. Med.* 8, 730243. 10.3389/fmed.2021.730243 PMC8678049 34926490

[DMM049707C19] Kitamoto, S., Alteri, C. J., Rodrigues, M., Nagao-Kitamoto, H., Sugihara, K., Himpsl, S. D., Bazzi, M., Miyoshi, M., Nishioka, T., Hayashi, A., et al. (2020). Dietary L-serine confers a competitive fitness advantage to Enterobacteriaceae in the inflamed gut. *Nat. Microbiol.* 5, 116-125. 10.1038/s41564-019-0591-6 31686025 PMC6925351

[DMM049707C20] Lucchini, V., Sivignon, A., Pieren, M., Gitzinger, M., Lociuro, S., Barnich, N., Kemmer, C. and Trebosc, V. (2021). The role of OmpR in bile tolerance and pathogenesis of adherent-invasive *Escherichia coli*. *Front. Microbiol.* 12, 684473. 10.3389/fmicb.2021.684473 34262546 PMC8273539

[DMM049707C21] Lyon, M. F. (1961). Gene action in the X-chromosome of the mouse (*Mus musculus* L.). *Nature* 190, 372-373. 10.1038/190372a0 13764598

[DMM049707C22] Martinez-Medina, M., Denizot, J., Dreux, N., Robin, F., Billard, E., Bonnet, R., Darfeuille-Michaud, A. and Barnich, N. (2014). Western diet induces dysbiosis with increased E coli in CEABAC10 mice, alters host barrier function favouring AIEC colonisation. *Gut* 63, 116-124. 10.1136/gutjnl-2012-304119 23598352

[DMM049707C23] Nadalian, B., Yadegar, A., Houri, H., Olfatifar, M., Shahrokh, S., Asadzadeh Aghdaei, H., Suzuki, H. and Zali, M. R. (2021). Prevalence of the pathobiont adherent-invasive *Escherichia coli* and inflammatory bowel disease: a systematic review and meta-analysis. *J. Gastroenterol. Hepatol.* 36, 852-863. 10.1111/jgh.15260 32929762

[DMM049707C24] Palmela, C., Chevarin, C., Xu, Z., Torres, J., Sevrin, G., Hirten, R., Barnich, N., Ng, S. C. and Colombel, J.-F. (2018). Adherent-invasive *Escherichia coli* in inflammatory bowel disease. *Gut* 67, 574-587. 10.1136/gutjnl-2017-314903 29141957

[DMM049707C25] Sasaki, M., Sitaraman, S. V., Babbin, B. A., Gerner-Smidt, P., Ribot, E. M., Garrett, N., Alpern, J. A., Akyildiz, A., Theiss, A. L., Nusrat, A. et al. (2007). Invasive *Escherichia coli* are a feature of Crohn's disease. *Lab. Invest.* 87, 1042-1054. 10.1038/labinvest.3700661 17660846

[DMM049707C26] Sevrin, G., Massier, S., Chassaing, B., Agus, A., Delmas, J., Denizot, J., Billard, E. and Barnich, N. (2020). Adaptation of adherent-invasive *E. coli* to gut environment: Impact on flagellum expression and bacterial colonization ability. *Gut Microbes* 11, 364-380. 10.1080/19490976.2017.1421886 29494278 PMC7524368

[DMM049707C27] Sivignon, A., de Vallée, A., Barnich, N., Denizot, J., Darcha, C., Pignède, G., Vandekerckove, P. and Darfeuille-Michaud, A. (2015a). *Saccharomyces cerevisiae* CNCM I-3856 prevents colitis induced by AIEC bacteria in the transgenic mouse model mimicking Crohn's disease. *Inflamm. Bowel Dis.* 21, 276-286. 10.1097/MIB.0000000000000280 25569734

[DMM049707C28] Sivignon, A., Yan, X., Alvarez Dorta, D., Bonnet, R., Bouckaert, J., Fleury, E., Bernard, J., Gouin, S. G., Darfeuille-Michaud, A. and Barnich, N. (2015b). Development of heptylmannoside-based glycoconjugate antiadhesive compounds against adherent-invasive *Escherichia coli* bacteria associated with Crohn's disease. *MBio* 6, e01298-15. 10.1128/mBio.01298-15 26578673 PMC4659459

[DMM049707C29] Sivignon, A., Yu, S.-Y., Ballet, N., Vandekerckove, P., Barnich, N. and Guerardel, Y. (2021). Heteropolysaccharides from S. cerevisiae show anti-adhesive properties against *E. coli* associated with Crohn's disease. *Carbohydr. Polym.* 271, 118415. 10.1016/j.carbpol.2021.118415 34364556

[DMM049707C30] Spalinger, M. R., Shawki, A., Chatterjee, P., Canale, V., Santos, A., Sayoc-Becerra, A., Scharl, M., Tremblay, M. L., Borneman, J. and McCole, D. F. (2022). Autoimmune susceptibility gene PTPN2 is required for clearance of adherent-invasive *Escherichia coli* by integrating bacterial uptake and lysosomal defence. *Gut* 71, 89-99. 10.1136/gutjnl-2020-323636 33563644 PMC8666829

[DMM049707C31] Viennois, E., Bretin, A., Dubé, P. E., Maue, A. C., Dauriat, C. J. G., Barnich, N., Gewirtz, A. T.and Chassaing, B. (2020). Dietary emulsifiers directly impact adherent-invasive *E. coli* gene expression to drive chronic intestinal inflammation. *Cell Rep.* 33, 108229. 10.1016/j.celrep.2020.108229 33027647 PMC7539532

[DMM049707C32] Zhang, S., Morgan, X. C., Dogan, B., Martin, F.-P., Strickler, S. R., Oka, A., Herzog, J., Liu, B., Dowd, S. E., Huttenhower, C. et al. (2022). Mucosal metabolites fuel the growth and virulence of *E. coli* linked to Crohn's disease. *JCI Insight* 7, e157013. 10.1172/jci.insight.157013 35413017 PMC9220930

