# Supplementary material for: An adherent-invasive Escherichia coli-colonized mouse model to evaluate microbiota-targeting strategies in Crohn's disease
Source: Dis Model Mech. 2022 Oct 24;15(10):dmm049707. doi: 10.1242/dmm.049707 (PMC9637268; doi:10.1242/dmm.049707)
Supplement: Supplementary information [file dmm-15-049707-s1.pdf]

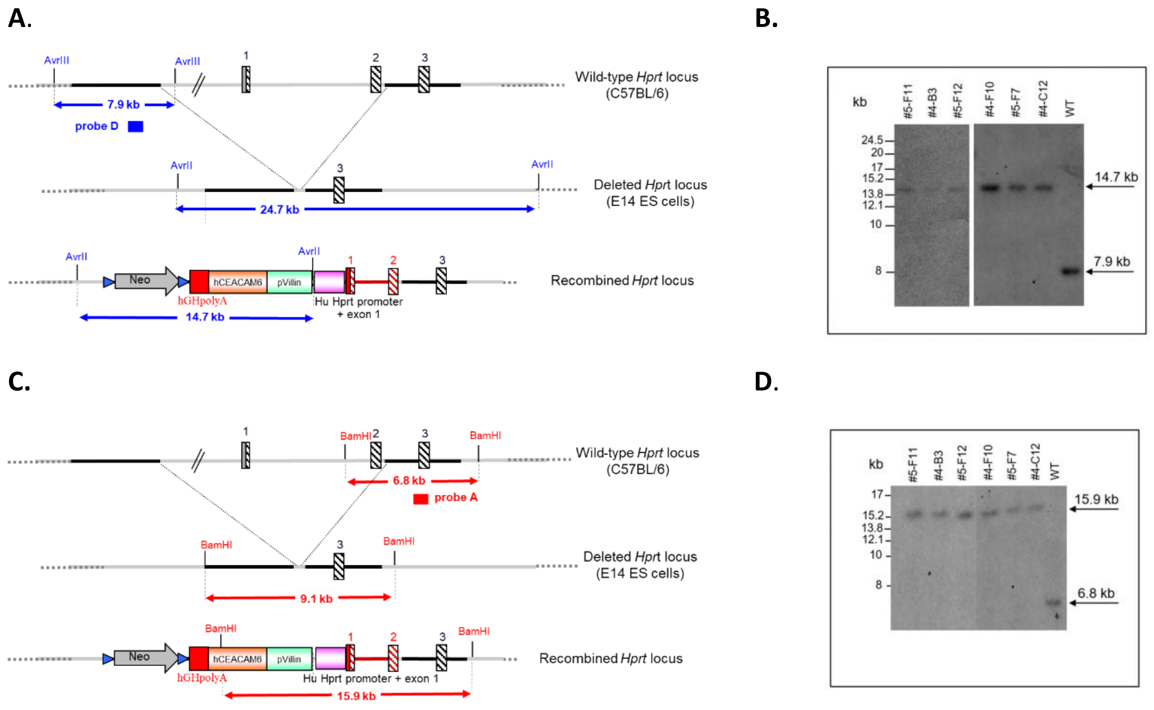

Fig. S1. Southern blot confirmation of homologous recombination events in HAT-resistant E14 embryo-derived stem (ES) cells. **A.** Schematic representation of the wild-type *Hprt* allele and of the recombined allele with the relevant restriction sites is shown (*related to figure 1*). The strategy for the 5’ Southern blot analysis is indicated. **B.** The genomic DNA of the tested ES cell clones was compared with wild-type DNA (C57BL/6). The digested DNA was blotted on a nylon membrane and hybridized with the 5’ probe D to detect the AvrII fragment. **C.** Schematic representation of the wild-type *Hprt* allele and the recombined allele with the relevant restriction sites is shown (*related to figure 1*). The strategy for the 3’ Southern blot analysis is indicated. **D.** The genomic DNA of the tested ES cell clones was compared with wild-type DNA (C57BL/6). The digested DNA was blotted on a nylon membrane and hybridized with the 3’ probe A to detect the BamHI fragment.

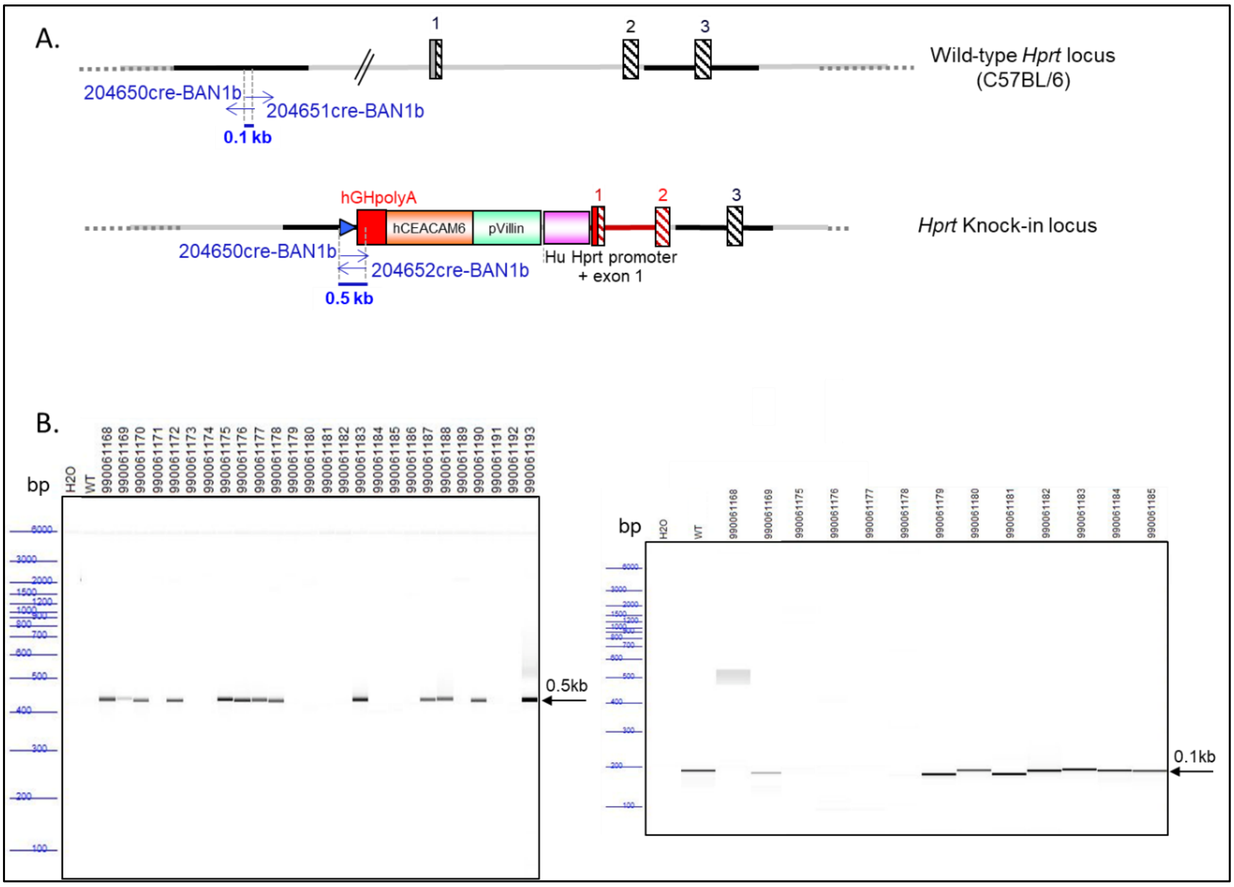

Fig. S2. Representative PCR results of the heterozygous females of interest. A. Schematic representation of the wild-type and neo-deleted knock-in *Hprt* loci (*related to figure 1*). Arrows indicate the primer binding sites. The primer sets and the size of the amplification products are indicated. **B.** The left panel shows the results obtained using the primer set to detect the neo-excised recombined *Hprt* allele with a 0.5 kb amplification product, and the right panel shows the results obtained using the primer set to detect the wild-type *Hprt* allele with a 0.1 kb amplification product. Samples containing wild-type genomic DNA (WT) or no DNA (H_20) served as positive and negative controls, respectively. PCR fragments were separated by capillary electrophoresis using an AATI ZAG^{TM} Fragment Analyzer and were analysed using PROSize 2.0 analytical software.

Disease Models & Mechanisms • Supplementary information

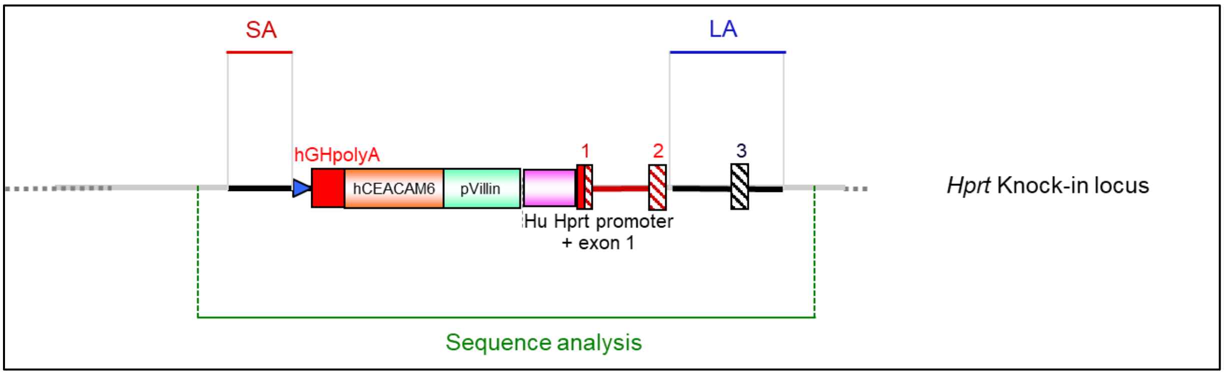

Fig. S3. Sequence analysis of the identified animals. Schematic representation of the sequenced knock-in locus, as well as a minimum of 1 kb downstream and upstream of both homology arms. SA: 5' short arm; LA: 3' long arm. Hatched black and red boxes represent murine and human Hprt exons, respectively. The human *CEACAM6* cDNA is depicted as an orange box. Green box represents the villin promoter and pink box represents the human Hprt promoter. The red box depicts hGHpolyA.

Disease Models & Mechanisms • Supplementary information

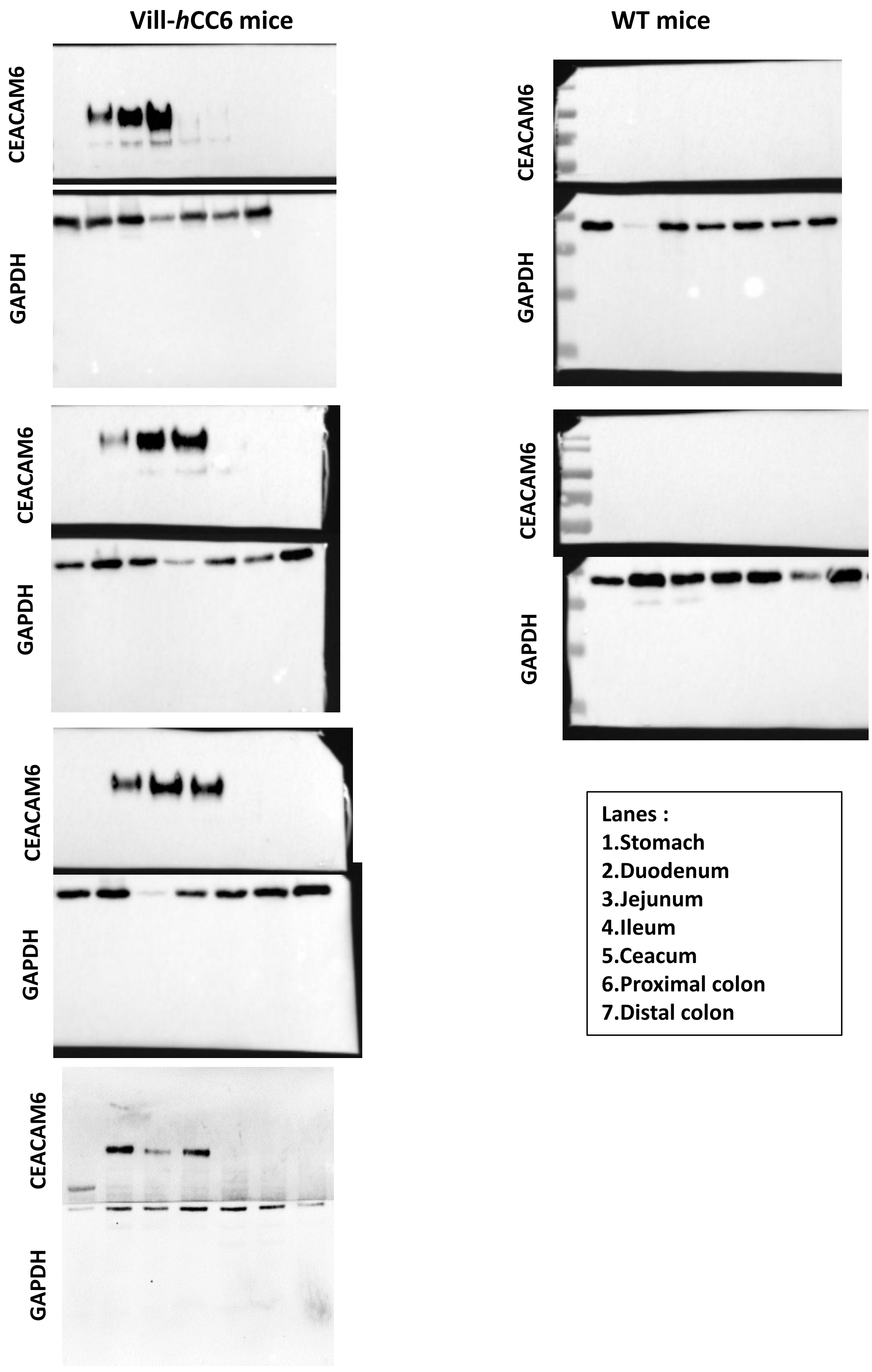

Fig. S4. CEACAM6 expression in the intestinal mucosa of WT or Vill-*h*CC6 mice. Uncropped western blots related to figure 3B from Vill-*h*CC6 and WT intestinal mucosa (n=4 for Vill-hCC6 and n=2 for WT mice).

Disease Models & Mechanisms • Supplementary information

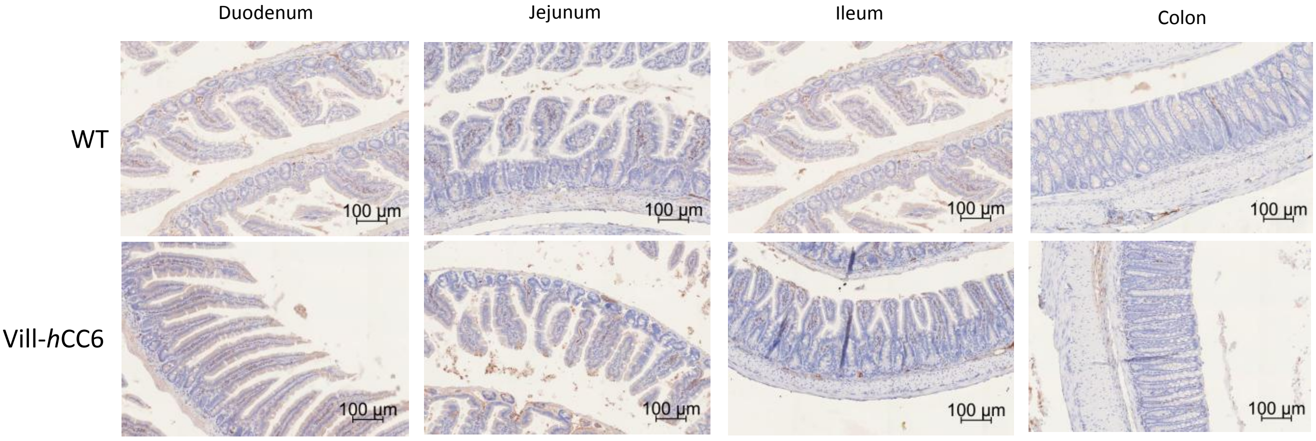

Fig. S5. Histological examinations of the intestinal mucosa of wild-type or Vill-hCC6 mice. Slides were scanned and assessed with an AxioScan Z1 using Zen 2.3 Pro software.

Disease Models & Mechanisms • Supplementary information

Table S1. Primers and PCR conditions for Vill-*h*CC6 mice genotyping

Primers sequences 5'-3'	bp	PCR conditions
***Hprt* wild-type allele**		
204650cre-BAN1b: TGTAACTTAGAACGTCAGTAGTCATAGGAACTGC<br>204651cre-BAN1b: AAAGAATCAATGCTTCCAGTGCTACCA	176	94°C 120s; (94°C 30s, 65°C 30s, 68°C 300s) x30; 68°C 480s Accuprime (Invitrogen)
***Hprt* Knock-in allele**		
204650cre-BAN1b: TGTAACTTAGAACGTCAGTAGTCATAGGAACTGC<br>204652cre-BAN1b: GTGGGACATTTGAGTTGCTTGCTTG	425	

Table S2. Score DAI (Disease Activity Index)

**Symptoms/**score	**Characteristics**
**Body weight loss**	
0	No loss
1	1-5 % loss of body weight
2	5-10 % loss of body weight
3	10-20 % loss of body weight
4	>20 % loss of body weight
**Stool consistency**	
0	Normal faeces
1	Loose stool
2	Watery diarrhea
3	Slimy diarrhea, little blood
4	Severe watery diarrhea with blood
**Blood in stool**	
0	No blood
2	Presence of blood
4	Visible bleeding

Disease Models & Mechanisms • Supplementary information
